# Progression of mild Alzheimer’s disease: knowledge and prediction models required for future treatment strategies

**DOI:** 10.1186/alzrt210

**Published:** 2013-10-07

**Authors:** Carina Wattmo, Åsa K Wallin, Lennart Minthon

**Affiliations:** 1 Clinical Memory Research Unit, Department of Clinical Sciences, Malmö, Lund University, Malmö, Sweden

## Abstract

**Introduction:**

Knowledge of longitudinal progression in mild Alzheimer’s disease (AD) is required for the evaluation of disease-modifying therapies. Our aim was to observe the effects of long-term cholinesterase inhibitor (ChEI) therapy in mild AD patients in a routine clinical setting.

**Methods:**

This was a prospective, open-label, non-randomized, multicenter study of ChEI treatment (donepezil, rivastigmine or galantamine) conducted during clinical practice. The 734 mild AD patients (Mini-Mental State Examination (MMSE) score 20 to 26) were assessed at baseline and then semi-annually over three years. Outcome measures included the MMSE, Alzheimer’s Disease Assessment Scale-cognitive subscale (ADAS-cog), Clinician’s Interview-Based Impression of Change (CIBIC) and Instrumental Activities of Daily Living (IADL) scale.

**Results:**

After three years of ChEI therapy, 31% (MMSE) and 33% (ADAS-cog) of the patients showed improved/unchanged cognitive ability, 33% showed improved/unchanged global performance and 14% showed improved/unchanged IADL capacity. Higher mean dose of ChEI and lower educational level were both predictors of more positive longitudinal cognitive and functional outcomes. Older participants and those with a better IADL score at baseline exhibited a slower rate of cognitive decline, whereas younger participants and those with higher cognitive status showed more preserved IADL ability over time. Gender and apolipoprotein E (APOE) genotype showed inconsistent results. Prediction models using the abovementioned scales are presented.

**Conclusions:**

In naturalistic mild AD patients, a marked deterioration in IADL compared with cognitive and global long-term outcomes was observed, indicating the importance of functional assessments during the early stages of the disease. The participants’ time on ChEI treatment before inclusion in studies of new therapies might affect their rate of decline and thus the comparisons of changes in scores between various studies. An increased understanding of expected disease progression in different domains and potential predictors of disease progression is essential for assessment of future therapies in AD.

## Introduction

Alzheimer’s disease (AD) is an insidiously progressive neurodegenerative disorder characterized by multiple cognitive deficiencies, including increasing impairment in memory, orientation, language and executive ability, as well as deterioration of functional capacity [1]. Currently, the main treatment for mild-to-moderate AD is acetylcholinesterase inhibitors (ChEI), which have shown positive symptomatic effects on cognition and function compared with a placebo in randomized clinical trials [2].

Novel ‘disease-modifying’ therapies attempt to block the course of AD in the early phase [3]. Because placebo-controlled clinical trials of more than six months duration in untreated individuals with AD are considered unethical, new longer studies are instead being conducted on patients who are already being treated with ChEI. Treatment strategies include various drug candidates that mainly aim to interfere with biological changes occurring in the brain during AD, such as the formation of beta-amyloid (Aβ) plaques. Hence, several research efforts, including active immunization with Aβ and passive immunization with humanized monoclonal anti-Aβ antibodies, are targeted towards preventing Aβ deposition. One study, in which AD patients were actively immunized with Aβ_42,_ reported clearance of amyloid plaques but no improvement in time to severe dementia or survival [4]. One explanation may be that immunotherapy is effective only in the mild stages of AD. Using pooled data from the two phase 3 passive immunization trials of solanezumab, the analyses demonstrated a significant slowing of cognitive decline and a trend towards slower deterioration in instrumental activities of daily living (IADL) after 80 weeks among individuals with mild but not moderate AD [5]. Furthermore, a six-month randomized trial of mild AD patients showed that souvenaid, a medical food product containing precursors and other specific nutrients required to enhance synapse formation, significantly increased memory performance in comparison with a placebo [6].

The majority of previous long-term extensions of clinical trials [7-9] and naturalistic studies of ChEI treatment in AD [10,11] have enrolled participants in both mild and moderate stages; hence, for this combined cohort, the cognitive, global and functional disease progression are well described. Few longitudinal studies have reported on progression in different domains exclusively in mild AD patients. This knowledge is essential to compare the trajectories of those who have received a new therapy in addition to ChEI with those of patients treated only with ChEI, especially since therapies that may modify disease progression in AD require thorough long-term evaluation.

In some clinical trials of immunotherapy, the treatment arms were stratified into presence/absence of the genetic risk factor apolipoprotein E (APOE) ϵ4 allele. Exploratory data analyses from the humanized monoclonal anti-Aβ antibody bapineuzumab trial observed that non-carriers of the APOE ϵ4 allele responded better cognitively than carriers [12]. Studies have shown contradicting results regarding the impact of the ϵ4 allele on cognitive response to ChEI treatment [13,14]. Possible predictors of disease progression and empirical models of the expected rate of change in cognition and IADL have not been analyzed exclusively in mild AD patients.

The aims of this study were to describe cognitive, global and functional longitudinal progression in mild AD, to identify the socio-demographic and clinical factors, such as APOE genotype that influence these outcomes, and to build prediction models based on data at the start of ChEI treatment.

## Methods

### Study and subjects

The Swedish Alzheimer Treatment Study (SATS) was undertaken to assess the longitudinal effectiveness of ChEI treatment (donepezil, rivastigmine or galantamine) over three years in AD patients in clinical practice. SATS is a prospective, open-label, observational, non-randomized, multicenter study that was reported at length in an earlier publication [10]. In total, 1,258 patients with predominantly mild-to-moderate AD were enrolled from 14 participating memory clinics across Sweden up until April 2008. Of these, all 734 individuals in the mild stage, that is, those participants with baseline Mini-Mental State Examination (MMSE) [15] scores ranging from 20 to 26, were included in the present analyses.

Outpatients aged 40 years and over who received a clinical diagnosis of dementia as defined by the *Diagnostic and Statistical Manual of Mental Disorders, 4*^*th*^*edition* (DSM-IV) [16] and had possible or probable AD according to the criteria of the National Institute of Neurological and Communicative Disorders and Stroke and the Alzheimer’s Disease and Related Disorders Association (NINCDS-ADRDA) [17] were considered for inclusion in SATS. Further inclusion criteria were that the participants had a knowledgeable caregiver, were living at home at the time of diagnosis and were assessable with MMSE at the start of ChEI treatment (baseline). Patients who did not fulfill the diagnostic criteria for AD, those already receiving active ChEI treatment or individuals with contraindications to ChEI were excluded from the study. Concomitant medications other than ChEIs were documented at baseline and permitted during the study, with the exception of memantine.

All patients and/or caregivers gave their written informed consent to participate in SATS, which was conducted according to the provisions of the Helsinki Declaration and was approved by the Ethics Committee of Lund University, Lund, Sweden.

The patients were evaluated in a well-structured follow-up program, which assessed cognition, global performance and functional capacity at the start of ChEI treatment, after two months (MMSE and global rating only), and semi-annually for a period of three years. After recruitment in SATS and evaluation at baseline, patients were prescribed ChEI according to the approved product recommendations. The choice of treatment (donepezil, rivastigmine or galantamine) was left entirely to the physician’s discretion and professional judgment. The ChEI dose was recorded after two months of treatment, and every six months after baseline assessment. Trained dementia nurses assessed the functional capacity of participants through caregiver interviews.

### Outcome measures

Cognitive ability was assessed using the MMSE scale, with a range from 0 to 30, in which a lower score indicates more impaired cognition, and using the Alzheimer’s Disease Assessment Scale-cognitive subscale (ADAS-cog) (0 to 70 points) [18], in which a lower score indicates better cognition. The Clinician Interview-Based Impression of Change (CIBIC) [19] was used as a global measure of ‘change from baseline’.

Ability to perform daily activities was assessed using the IADL scale [20] that consists of the following eight items: ability to i) use the telephone, ii) go shopping, iii) prepare food, iv) undertake housekeeping activities, v) do laundry, vi) travel independently, vii) be responsible for own medications and viii) handle finances. Each item was scored from 1 (no impairment) to 3 to 5 (severe impairment), allowing a total range of 8 to 31 points. Some of the instrumental tasks might be gender-dependent among the older generation. Therefore, a mathematical correction of the sum of the IADL scores was performed, to prevent those tasks from affecting the results. The formula used the data from the rated items to estimate a total score within the range of the total IADL scale [21].

**Figure 1:**
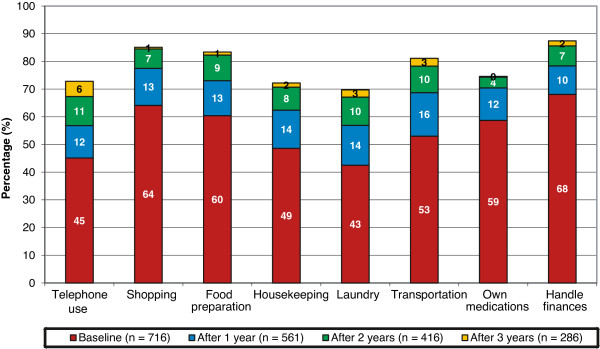
**IADL, dependence in IADL items.** Percentage of mild AD patients who are not able to perform individual IADL tasks independently (IADL score 2 to 5) at baseline and after one, two and three years of ChEI treatment. AD, Alzheimer’s disease; ChEI, cholinesterase inhibitor; IADL, Instrumental Activities of Daily Living.

For each assessment, mean MMSE, ADAS-cog and IADL changes from baseline with 95% confidence intervals (CI) were calculated. To facilitate comparisons among these scales, changes in the scores calculated as positive values should be interpreted as indicating improvement and those calculated as negative values interpreted as indicating decline. Percentages of improved/unchanged patients, pre-defined as those who showed an improvement or no change (≥0 points difference) at the respective assessment, were also calculated for the MMSE, ADAS-cog and IADL scales. The assessments of change in global performance from the start of ChEI treatment were made at all intervals using a 7-point scale that varies from 1 (very much improved) to 7 (marked worsening), with 4 indicating no change. No guidelines or descriptors were provided to define the individual ratings. The classification between, for example, minimally improved or very much improved was left to the physician’s clinical judgment. In Figure 1, the patients who could not perform an individual IADL task independently (IADL score 2 to 5) were categorized as ‘need assistance’.

### Statistical analyses

The IBM Statistical Package for the Social Sciences (SPSS) software (version 21.0; IBM Corporation, Armonk, NY, USA) was used to perform statistical analyses. The level of significance was defined as *P* <0.05 if not otherwise specified. Observed-case analyses were performed to avoid overestimation of the treatment effect by imputing higher, earlier outcome scores in a longitudinal study of a progressively deteriorating disease. Parametric tests were used because of the large sample and the approximately normally distributed continuous potential predictors. A *t*-test was used to compare the differences between the means obtained for two independent groups, and a χ^2^ test was computed to analyze categorical variables. Pearson’s correlation coefficient was calculated to investigate any linear associations between continuous variables.

Mixed, linear and non-linear fixed and random coefficient regression models [22] using the individual as a hierarchical variable (that is, to allow intra-individual correlation) were performed. In addition, the mixed-effects models take into consideration the varying number of assessment information available for each patient and unequal time intervals between follow-up visits, which are common statistical limitations in long-term studies. The individuals who dropped out during the study contributed information during the time of participation; thus, we considered the trajectories of all patients.

Time was defined as the exact number of months between the start of ChEI treatment and each assessment, which indicated that all data points were used at the actual time intervals. To adjust for baseline differences, the initial cognitive or functional scores for each individual and their interaction with linear and quadratic terms for months in the study (to enable a non-linear rate of change in the models) were included as fixed effects, that is, time in months (or time in months^2^) × MMSE (ADAS-cog or IADL) baseline score. Hence, the dependent variables were the cognitive or functional scores assigned at the second and subsequent evaluations for each patient; thus, the mixed-effects models do not intend to predict the scores at the start of ChEI treatment. The random terms in the models were an intercept and time in months, with a variance components covariance matrix. Several socio-demographic and clinical characteristics were included as fixed effects. The potential predictors analyzed were classical risk factors, such as age at the start of ChEI therapy (in years), the clinician’s estimate of age at onset (in years), gender, years of education, whether a carrier of the APOE ϵ4 allele (no/yes), whether living alone (no/yes), number of medications at baseline and whether taking specific concomitant medications (no/yes for each group) including antihypertensive/cardiac therapy, anti-diabetics, lipid-lowering agents, estrogens, non-steroidal anti-inflammatory drugs (NSAIDs)/acetylsalicylic acid, antidepressants, antipsychotics and anxiolytics/sedatives/hypnotics. The effect of ChEI treatment was analyzed using different drug agents and dosages. Lastly, some biologically plausible interactions with disease severity at the start of treatment or with time in the study were included in the mixed-effects models; these were gender, education, age and APOE genotype.

The ChEI drugs were coded as a set of dummy variables. The dose could vary during the treatment period for an individual patient and between patients. For that reason, the mean dose used during the entire follow-up period was calculated for each patient. To obtain a similar metric for percentage maximum dosage for each of the three ChEI drugs, the mean dose was divided by the maximum recommended dose for each agent, that is, 10 mg for donepezil, 12 mg for rivastigmine (oral therapy) and 24 mg for galantamine. The term ‘type of ChEI × dose’ was also included in the models. Non-significant variables (*P* >0.05) were removed in a backward stepwise elimination manner. The hierarchical principle was applied in these analyses; terms that appeared in interactions were not considered for elimination.

## Results

### Baseline characteristics

The socio-demographic and clinical characteristics of the 734 mild AD patients are shown in Table 1. Male patients had significantly more years of education (mean ± standard deviation (SD), 10.2 ± 3.0 vs 9.3 ± 2.4 years; t(730) = 4.12; *P* <0.001) and a lower IADL capacity at the start of ChEI treatment (15.4 ± 5.1 vs 14.3 ± 5.0 points; t(714) = 2.94; *P* = 0.003) compared with females. No significant differences in age, cognitive ability or number of concomitant medications at baseline were found between genders.

**Table 1:** **Socio-demographic and clinical characteristics (*n *** **= 734)**

**Variable**	
Female gender	473 (64%)
APOE ϵ4 carrier, (*n* = 718)	493 (69%)
Solitary living at baseline	267 (36%)
Anti-hypertensives/Cardiac therapy	290 (40%)
Anti-diabetics	38 (5%)
Lipid-lowering agents	94 (13%)
Estrogens	52 (7%)
NSAIDs/Acetylsalicylic acid	221 (30%)
Anti-depressants	183 (25%)
Anti-psychotics	26 (4%)
Anxiolytics/Sedatives/Hypnotics	111 (15%)
Variable	**Mean ± standard deviation**
Estimated age at onset (years)	72.3 ± 7.1
Estimated duration of AD at baseline (years)	2.9 ± 2.0
Age at first assessment (years)	75.2 ± 6.8
Education (years)	9.6 ± 2.6
MMSE score at baseline	23.4 ± 2.0
ADAS-cog score (0–70) at baseline	17.5 ± 6.7
IADL score at baseline	14.7 ± 5.0
Number of concomitant medications at baseline	2.9 ± 2.4

ADAS-cog, Alzheimer’s Disease Assessment Scale-cognitive subscale; APOE, apolipoprotein E; IADL, Instrumental Activities of Daily Living scale; MMSE, Mini-Mental State Examination; NSAIDs, non-steroidal anti-inflammatory drugs.

Individuals carrying the APOE ϵ4 allele (69%) were younger on average (74.4 ± 6.9 vs 76.6 ± 6.5 years; t(716) = 4.09; *P* <0.001), had an earlier onset of AD (71.4 ± 7.2 vs 73.9 ± 6.7 years; t(713) = 4.50; *P* <0.001) and had a higher IADL capacity at baseline (14.3 ± 5.1 vs 15.3 ± 4.9 points; t(698) = 2.57; *P* = 0.010) compared with non-carriers. No differences in years of education, cognitive ability or number of concomitant medications at the start of ChEI treatment were detected between carriers and non-carriers of the APOE ϵ4 allele.

Older age at baseline was linearly associated with lower IADL capacity (*n* = 716, *r* = 0.326, *P* <0.001) and greater number of concomitant medications at baseline (*n* = 734, *r* = 0.228, *P <*0.001). In addition, lower IADL capacity and more concomitant medications correlated weakly (*n* = 716, *r* = 0.194, *P <*0.001) with each other. Weak linear relationships between older age and lower level of education (*n* = 732, *r* = −0.130, *P <*0.001) or more impaired cognition (MMSE: *n* = 734, *r* = −0.097, *P =* 0.008; ADAS-cog: *n* = 724, *r* = 0.181, *P* <0.001) at the start of ChEI therapy were found.

### Longitudinal outcomes in mild AD

**Figure 2:**
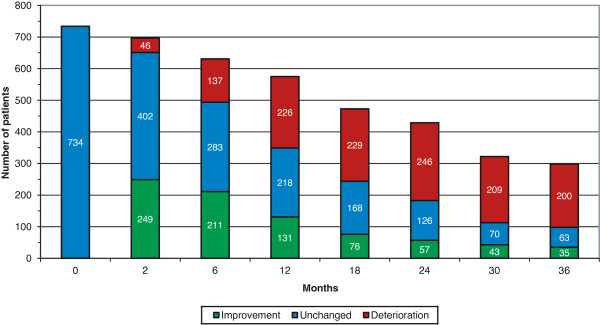
**CIBIC, changes in global performance.** The changes in global performance (CIBIC) from the start of ChEI treatment over three years in mild AD. CIBIC score 1 to 3 was considered as improvement, 4 as unchanged and 5 to 7 as deterioration. Among the remaining patients in the study, 61% exhibited global improvement or no changes after one year, 43% after two years and 33% after three years of ChEI therapy. AD, Alzheimer’s disease; ChEI, cholinesterase inhibitor; CIBIC, Clinician’s Interview-Based Impression of Change.

The mean MMSE, ADAS-cog and IADL actual scores and the changes from baseline scores during three years are shown in Table 2. The percentages of patients who showed improvement or remained unchanged at each visit according to these measures are also reported. In Table 3, the above-mentioned results are presented separately for APOE ϵ4-carriers and non-carriers. The changes in global performance (CIBIC) from the start of ChEI treatment over the three-year study are illustrated in Figure 2. After one year, 23% of the remaining SATS participants with mild AD were globally improved and 38% unchanged, after two years, the proportions were 13% and 29%, respectively, and after three years, the proportions were 12% and 21%. The percentage of patients who needed help to perform individual IADL items is displayed in Figure 1. At baseline, 45% to 65% could not perform usual IADL tasks independently, and after three years 70% to 85% of the remaining mild AD patients in the study needed assistance with IADLs.

**Table 2:** Changes in cognitive and functional abilities during three years of ChEI treatment in mild AD

**Variable**	**2 months**	**6 months**	**12 months**	**18 months**	**24 months**	**30 months**	**36 months**
	**(*n *** **= 706)**	**(*n *** **= 662)**	**(*n *** **= 597)**	**(*n *** **= 521)**	**(*n *** **= 450)**	**(*n *** **= 374)**	**(*n *** **= 306)**
Completion rate (%)	96.2	90.2	81.3	71.0	61.3	51.0	41.7
MMSE score^a^	24.0 (23.8, 24.2)	23.5 (23.3, 23.8)	22.8 (22.5, 23.2)	22.1 (21.7, 22.5)	21.4 (20.9, 21.8)	21.1 (20.5, 21.6)	20.6 (20.0, 21.2)
ADAS-cog (0 to 70) score^a^		17.4 (16.8, 18.0)	18.3 (17.6, 19.0)	19.5 (18.6, 20.3)	20.1 (19.1, 21.1)	21.3 (20.0, 22.7)	21.7 (20.2, 23.2)
IADL score^a^		15.8 (15.4, 16.2)	17.0 (16.6, 17.5)	17.9 (17.4, 18.5)	18.8 (18.2, 19.4)	19.4 (18.7, 20.1)	19.8 (19.1, 20.6)
MMSE score, change from baseline^a^	0.61 (0.43, 0.78)	0.19 (−0.04, 0.42)	−0.57 (−0.86, −0.28)	−1.31 (−1.66, −0.96)	−2.20 (−2.64, −1.77)	−2.54 (−3.06, −2.02)	−3.10 (−3.66, −2.54)
ADAS-cog (0 to 70) score, change from baseline^a^		−0.15 (−0.65, 0.34)	−1.20 (−1.77, −0.63)	−2.58 (−3.27, −1.90)	−3.81 (−4.59, −3.03)	−4.97 (−6.09, −3.85)	−6.12 (−7.35, −4.89)
IADL score, change from baseline^a^		−1.12 (−1.34, −0.91)	−2.48 (−2.78, −2.17)	−3.64 (−4.01, −3.26)	−4.75 (−5.18, −4.33)	−5.54 (−6.06, −5.02)	−6.27 (−6.86, −5.68)
MMSE score, improved/unchanged patients (%)	72.7	64.1	55.0	46.9	41.9	38.2	33.0
ADAS-cog (0 to 70) score, improved/unchanged patients (%)		55.3	48.8	41.8	36.7	32.9	31.1
IADL score, improved/unchanged patients (%)		49.7	34.2	24.3	18.0	14.8	13.8

^a^Mean (95% confidence interval).

For clarity, clinical improvements for all scales have been tabulated as positive changes from the start of ChEI treatment (baseline).

ADAS-cog, Alzheimer’s Disease Assessment Scale-cognitive subscale; ChEI, cholinesterase inhibitor; IADL, Instrumental Activities of Daily Living scale; MMSE, Mini-Mental State Examination.

**Table 3:** Changes in scores during three years of ChEI treatment in mild AD by APOE genotype

	**MMSE score**^**a**^			**MMSE score, change from baseline**^**a**^			**MMSE score, improved/unchanged patients (%)**		
**Number of non-carriers/ϵ4-carriers**^**b**^	**Non-carriers**	**ϵ4-carriers**	***P*- value**	**Non-carriers**	**ϵ4-carriers**	***P*-value**	**Non-carriers**	**ϵ4-carriers**	***P*-value**
Baseline (*n* = 225/493)	23.3 (23.0, 23.5)	23.4 (23.2, 23.6)	0.326						
2 months (*n* = 213/469)	24.0 (23.6, 24.4)	24.0 (23.8, 24.3)	0.843	0.64 (0.28, 1.00)	0.60 (0.39, 0.80)	0.834	73.2	72.7	0.926
6 months (*n* = 194/423)	23.5 (23.0, 23.9)	23.5 (23.2, 23.9)	0.864	0.20 (−0.20, 0.59)	0.17 (−0.12, 0.46)	0.920	61.9	65.2	0.417
12 months (*n* = 178/388)	22.7 (22.1, 23.3)	22.9 (22.5, 23.3)	0.597	−0.53 (−1.10, 0.04)	−0.56 (−0.89, −0.22)	0.945	56.2	54.4	0.716
18 months (*n* = 145/320)	21.7 (20.9, 22.5)	22.3 (21.9, 22.8)	0.189	−1.53 (−2.29, −0.78)	−1.18 (−1.57, −0.80)	0.420	49.0	46.3	0.617
24 months (*n* = 130/292)	21.0 (20.0, 22.0)	21.5 (21.0, 22.1)	0.361	−2.52 (−3.45, −1.60)	−2.05 (−2.54, −1.57)	0.377	44.6	40.8	0.522
30 months (*n* = 97/219)	21.5 (20.5, 22.5)	20.9 (20.2, 21.6)	0.342	−2.04 (−3.03, −1.05)	−2.74 (−3.36, −2.11)	0.230	47.4	34.7	0.034
36 months (*n* = 91/199)	21.0 (19.9, 22.2)	20.3 (19.6, 21.1)	0.325	−2.81 (−3.83, −1.80)	−3.26 (−3.95, −2.57)	0.471	35.2	31.7	0.591
	**ADAS-cog (0 to 70) score**^**a**^			**ADAS-cog (0 to 70) score, change from baseline**^**a**^			**ADAS-cog (0 to 70) score, improved/unchanged patients (%)**		
Number of non-carriers/ϵ4-carriers^b^	**Non-carriers**	**ϵ4-carriers**	**P-value**	**Non-carriers**	**ϵ4-carriers**	***P*-value**	**Non-carriers**	**ϵ4-carriers**	***P*-value**
Baseline (*n* = 220/488)	17.8 (17.0, 18.6)	17.1 (16.6, 17.7)	0.220						
6 months (*n* = 187/414)	16.8 (15.9, 17.8)	17.6 (16.8, 18.3)	0.277	0.84 (0.09, 1.58)	−0.57 (−1.22, 0.08)	0.011	63.0	51.5	0.010
12 months (*n* = 171/376)	18.1 (16.8, 19.4)	18.5 (17.6, 19.3)	0.666	0.06 (−0.84, 0.96)	−1.78 (−2.50, −1.05)	0.004	57.7	44.5	0.005
18 months (*n* = 137/306)	19.4 (17.7, 21.1)	19.5 (18.5, 20.5)	0.893	−1.88 (−3.30, −0.47)	−2.89 (−3.68, −2.11)	0.185	48.1	38.8	0.075
24 months (*n* = 117/274)	20.4 (18.2, 22.5)	20.1 (18.9, 21.3)	0.812	−3.34 (−4.98, −1.70)	−4.07 (−4.96, −3.18)	0.409	43.5	33.3	0.065
30 months (*n* = 92/203)	20.7 (18.3, 23.1)	21.7 (20.1, 23.3)	0.526	−3.71 (−5.85, −1.58)	−5.54 (−6.87, −4.22)	0.139	42.2	28.7	0.031
36 months (*n* = 85/180)	20.9 (18.2, 23.5)	22.2 (20.3, 24.0)	0.427	−4.96 (−7.24, −2.68)	−6.78 (−8.28, −5.29)	0.181	40.5	26.8	0.032
	**IADL score**^**a**^			**IADL score, change from baseline**^**a**^			**IADL score, improved/unchanged patients (%)**		
Number of non-carriers/ϵ4-carriers^b^	**Non-carriers**	**ϵ4-carriers**	***P*-value**	**Non-carriers**	**ϵ4-carriers**	***P*-value**	**Non-carriers**	**ϵ4-carriers**	***P*-value**
Baseline (*n* = 219/481)	15.3 (14.7, 16.0)	14.3 (13.8, 14.7)	0.010						
6 months (*n* = 189/413)	16.3 (15.6, 17.0)	15.5 (15.0, 16.0)	0.081	−1.07 (−1.45, −0.69)	−1.14 (−1.41, −0.88)	0.758	48.1	50.6	0.597
12 months (*n* = 175/379)	17.6 (16.8, 18.4)	16.8 (16.2, 17.4)	0.123	−2.31 (−2.80, −1.82)	−2.55 (−2.93, −2.16)	0.482	34.3	34.8	0.923
18 months (*n* = 142/311)	18.6 (17.6, 19.6)	17.6 (16.9, 18.3)	0.099	−3.64 (−4.29, −2.99)	−3.63 (−4.09, −3.16)	0.974	26.6	23.4	0.477
24 months (*n* = 128/283)	19.8 (18.7, 20.9)	18.4 (17.7, 19.1)	0.029	−5.13 (−5.96, −4.29)	−4.60 (−5.10, −4.10)	0.268	18.4	17.9	0.889
30 months (*n* = 95/216)	19.9 (18.7, 21.1)	19.1 (18.3, 19.9)	0.273	−5.11 (−6.07, −4.15)	−5.71 (−6.34, −5.09)	0.301	16.3	14.4	0.728
36 months (*n* = 91/191)	20.3 (18.9, 21.7)	19.7 (18.8, 20.6)	0.500	−6.23 (−7.31, −5.14)	−6.31 (−7.03, −5.60)	0.895	17.0	12.1	0.266

^a^Mean (95% confidence interval).

^b^No difference in dropout was found between APOE ϵ4-carriers and non-carriers, χ^2^(1) = 0.148, *P* = 0.700.

For clarity, clinical improvements for all scales have been tabulated as positive changes from the start of ChEI treatment (baseline).

ADAS-cog, Alzheimer’s Disease Assessment Scale-cognitive subscale; APOE, apolipoprotein E; ChEI, cholinesterase inhibitor; IADL, Instrumental Activities of Daily Living scale; MMSE, Mini-Mental State Examination.

#### ChEI therapy

Of the 734 patients, 354 (48%) received donepezil, 162 (22%) rivastigmine and 218 (30%) galantamine. After one year, the mean ± SD doses of donepezil, rivastigmine and galantamine were 7.7 ± 2.6, 7.6 ± 2.9 and 18.4 ± 4.4 mg, respectively. After two years, they were 8.1 ± 2.5, 8.1 ± 2.9 and 19.4 ± 4.5 mg, and after three years the doses were 8.4 ± 2.4, 8.3 ± 2.7 and 20.3 ± 4.2 mg, respectively.

#### Dropout analyses

Overall, 58% of the mild AD patients did not complete the three-year study. The reasons for dropout from the study were admission to nursing home (12%, n = 87), side effects (8%, n = 57), initiation of concomitant memantine therapy (6%, n = 42), compliance problems (5%, n = 38), death (5%, n = 35), withdrawal of informed consent (5%, n = 35), switching to another study (4%, n = 31), poor effect/deterioration (4%, n = 28), somatic disease assumed unrelated to AD (3%, n = 19), switching to another ChEI agent (2%, n = 15), and other reasons (5%, n = 37).

The patients who remained in SATS for the complete three-year follow-up period exhibited significantly better cognitive (mean ± SD; MMSE, 23.6 ± 2.0 vs 23.1 ± 1.9 points; t(732) = 3.62; *P* <0.001; ADAS-cog, 16.1 ± 6.8 vs 18.4 ± 6.5 points; t(722) = −4.74; *P* <0.001) and IADL (13.5 ± 4.6 vs 15.5 ± 5.2 points; t(714) = −5.48; *P* <0.001) abilities at baseline than patients who dropped out, and received a greater percentage of the maximum recommended ChEI dose during the study (69 ± 17 vs 59 ± 18%; t(732) = 7.50; *P* <0.001). The other patient characteristics of gender, presence of the APOE ϵ4 allele, solitary living, age at onset, age at the start of ChEI treatment, years of education, number of concomitant medications and specific medications received did not differ between the completers and those who discontinued the study.

### Predictors of longitudinal cognitive and functional outcomes

In the mixed-effects models, only patients with three or more assessments (n = 656, 89.4%) were included, in order to enable analyses of a non-linear rate of cognitive or functional change. The models were performed (3,280 data points) to identify the socio-demographic and clinical factors that affected the long-term trajectories of mild AD patients. The percentages of variance that accounted for the dependent variable, regarding all fixed predictors, were 28.6% for MMSE, 37.6% for ADAS-cog, and 60.9% for IADL, indicating a moderate to very good fit of the models (*P* <0.001). The mixed-effects models, significant predictors, and unstandardized β coefficients with 95% CI are presented in Table 4. Male individuals (MMSE only), non-carriers of the APOE ϵ4 allele (ADAS-cog only), those with more preserved IADL ability at baseline and patients receiving a higher mean dose of ChEI during the study (irrespective of drug agent) exhibited a more favorable longitudinal cognitive outcome. Individuals living with a family member, those with better cognitive status at baseline and patients receiving a higher ChEI dose showed less deterioration in IADL during the three-year study.

**Table 4:** Factors affecting the long-term outcome with MMSE, ADAS-cog or IADL score as dependent variables

	**MMSE**		**ADAS-cog**		**IADL**	
Percentage of variance accounted for, all fixed terms	28.6%, *P* <0.001		37.6%, *P* <0.001		60.9%, *P* <0.001	
Significant predictors in final mixed models^a^	β (95% CI)	*P*-value	β (95% CI)	*P*-value	β (95% CI)	*P*-value
Fixed terms						
Intercept	−25.751	0.022	−14.101	0.048	0.808	0.728
	(−47.709, −3.793)		(−28.099, −0.102)		(−3.747, 5.363)	
Time in months from baseline	−0.924	<0.001	0.480	0.019	0.154	<0.001
	(−1.201, −0.648)		(0.078, 0.881)		(0.069, 0.239)	
Baseline assessment score	2.198	<0.001	1.696	<0.001	1.530	<0.001
	(1.272, 3.124)		(0.931, 2.462)		(1.262, 1.798)	
Baseline assessment score^2^		ns		ns	−0.021	<0.001
					(−0.029, −0.013)	
Time in months × Baseline assessment score	0.028	<0.001	0.014	<0.001	−0.001	0.512
	(0.019, 0.036)		(0.008, 0.021)		(−0.005, 0.003)	
Time in months^2^ × Baseline assessment score	−0.00007	<0.001	0.0002	0.002	−0.00006	0.043
	(−0.0001, −0.00004)		(0.00006, 0.0003)		(−0.0001, −0.000002)	
*Background variables:*						
Gender (male = 0, female = 1)	−0.582	0.001		ns		ns
	(−0.233, −0.931)					
APOE ϵ4 carrier (no = 0, yes = 1)		ns	1.278	0.015		ns
			(2.307, 0.249)			
Solitary living (no = 0, yes = 1)		ns		ns	0.776	0.001
					(1.253, 0.299)	
Education (years)	0.089	0.011	−0.111	0.281	−0.051	0.328
	(0.021, 0.158)		(−0.312, 0.090)		(−0.152, 0.051)	
Time in months × Education, years	−0.010	0.002	0.016	0.017	0.011	0.001
	(−0.016, −0.004)		(0.003, 0.028)		(0.005, 0.018)	
Age at first assessment (years)	0.491	0.001	0.285	0.002	0.042	0.023
	(0.202, 0.780)		(0.101, 0.469)		(0.006, 0.078)	
Time in months × Age	0.004	0.003	−0.009	<0.001		ns
	(0.001, 0.006)		(−0.014, −0.004)			
Baseline assessment score × Age	−0.022	<0.001	−0.015	0.004		ns
	(−0.034, −0.010)		(−0.025, −0.005)			
IADL score at baseline	−0.098	<0.001	0.225	<0.001	na	
	(−0.135, −0.062)		(0.117, 0.332)			
MMSE score at baseline	na		na		−0.238	<0.001
					(−0.358, −0.118)	
ChEI dose^b^	0.010	0.049	−0.044	0.002	−0.013	0.048
	(0.00004, 0.019)		(−0.071, −0.017)		(−0.026, −0.0001)	
*Random terms (variance)*						
Intercept	2.097	<0.001	17.298	<0.001	5.539	<0.001
	(1.647, 2.669)		(13.849, 21.606)		(4.473, 6.858)	
Time in months	0.026	<0.001	0.086	<0.001	0.027	<0.001
	(0.022, 0.031)		(0.071, 0.104)		(0.022, 0.032)	

Age at onset, number of medications and specific concomitant medications used at baseline and the variable comparing the ChEI agents were not significant.

β values were unstandardized and are expressed per one unit increase for continuous variables and for the condition present in dichotomous variables.

^a^Baseline assessment score = MMSE, ADAS-cog or IADL, respectively.

^b^Mean percentage of the maximum recommended dose, that is, 10 mg for donepezil, 12 mg for rivastigmine and 24 mg for galantamine.

*Abbreviations:* ADAS-cog, Alzheimer’s Disease Assessment Scale-cognitive subscale; APOE, apolipoprotein E; ChEI*,* cholinesterase inhibitors; CI, confidence interval; IADL, Instrumental Activities of Daily Living; MMSE*,* Mini-Mental State Examination; na not applicable; ns not significant.

The interaction effects of cognitive severity, age at baseline, time in months from the start of ChEI therapy, and years of education showed that these variables cannot be interpreted separately. Older individuals with mild AD exhibited a better long-term cognitive outcome compared with younger individuals. For example, 85-year-old patients with a baseline MMSE score of 23 demonstrated, on average, 2.3 points better than 65-year-old patients. Similarly, 85-year-old patients with a baseline ADAS-cog score of 18 showed an additional 6.4 points better compared with 65-year-old patients after three years of ChEI treatment. In contrast, the corresponding mean outcome in IADL score was 0.8 points more favorable for a 65-year-old vs an 85-year-old individual.

Furthermore, there was an interaction effect between years of education and time in the study. A higher level of education implied increased cognitive and functional impairments over time. For example, a patient with 15 years of education exhibited an additional 1.6 points of MMSE, 2.7 points of ADAS-cog and 2.2 points of IADL mean deterioration after 3 years compared with an individual with 9 years of education.

If not otherwise specified, the arbitrary examples presented in these calculations were based on an average patient that was aged 75 years, was a carrier of the APOE ϵ4 allele, living with a family member, had 10 years of education, exhibited an MMSE score of 23, ADAS-cog score of 18, IADL score of 15, and received 65% of the maximum recommended dose of ChEI.

The background variables age at onset, number of medications and specific concomitant medications, type of ChEI agent, type of ChEI × dose and the interaction effects of gender or APOE genotype with disease severity or time in the study were not significant when included in the mixed-effects models.

#### Prediction models

Non-linear regression models for calculation of the predicted MMSE, ADAS-cog or IADL score for a cohort of ChEI-treated mild AD patients, based on the respective baseline score are provided. These equations intend to predict the scores at subsequent assessments over a three-year period. The models explained a substantial degree of variance in the data set, that is, demonstrated a good fit, MMSE: (R^2^ = 0.261, R = 0.511, *P* <0.001), ADAS-cog: (R^2^ = 0.385, R = 0.621, *P* <0.001), and IADL: (R^2^ = 0.592, R = 0.769, *P* <0.001).

Predicted MMSE score:

$$\begin{array}{l} \hat{Y}=7.6014-\left(0.4374\times t\right)+\left(0.7116\times x_{i}\right)\\ \;+\left(0.0138\times {\mathrm{tx}}_{i}\right) \end{array}$$

Predicted ADAS-cog score:

$$\begin{array}{l} \hat{Y}=2.7835-\left(0.0537\times t\right)\\ \;+\left(0.8867\times x_{i}\right)-\left(0.0073\times x_{i}{}^{2}\right)+\left(0.0161\times {\mathrm{tx}}_{i}\right) \end{array}$$

Predicted IADL score:

$$\begin{array}{l} \hat{Y}=-6.1756+\left(0.3140\times t\right)\\ \;+\left(1.8752\times x_{i}\right)-\left(0.0023\times t^{2}\right)-\left(0.0299\times x_{i}{}^{2}\right)-\left(0.0032\times {\mathrm{tx}}_{i}\right) \end{array}$$

Where t = time in months between the baseline score and the actual visit, x_i_ = baseline MMSE (ADAS-cog or IADL) score.

**Figure 3:**
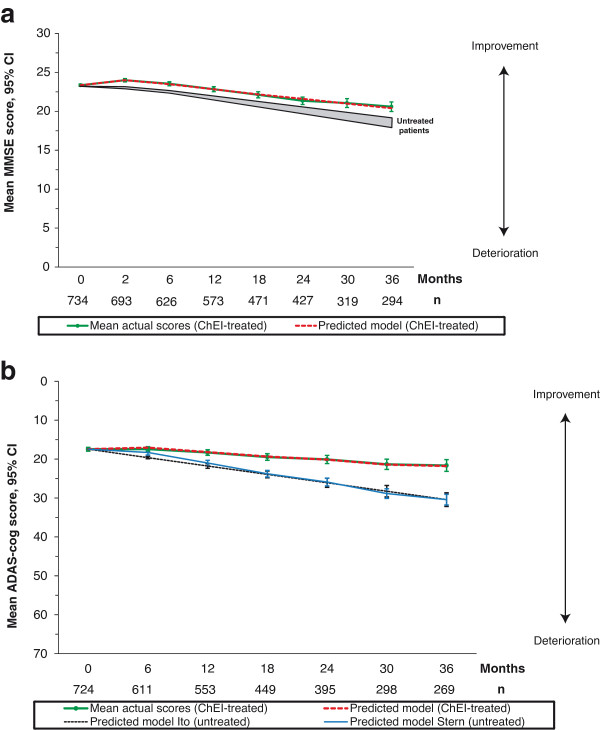
**Prediction of cognitive outcome during three years of ChEI treatment. a)** Predicted MMSE mean scores calculated from our non-linear regression model of ChEI-treated mild AD patients. The participants’ actual mean scores with 95% CI are also presented in the figure. The shaded area shows the decline of 1.4 to 1.8 MMSE points/year based on historical untreated individuals with mild AD [23]. **b)** Predicted ADAS-cog mean scores calculated from our non-linear regression model of ChEI-treated mild AD patients. Their actual mean scores with 95% CI are also presented in the figure. The mean predicted scores with 95% CI from two baseline-dependent prediction models of untreated AD patients are also included in the figure for comparison [24,25]. AD, Alzheimer’s disease; ADAS-cog, Alzheimer’s Disease Assessment Scale-cognitive subscale; ChEI, cholinesterase inhibitor; MMSE, Mini-Mental State Examination.

The MMSE and ADAS-cog prediction models are illustrated in Figure 3a, b, respectively. The SATS patients’ actual mean scores with 95% CI are also presented in the figures, together with expected decline in untreated individuals. Regarding MMSE, a reported deterioration of 1.4 to 1.8 points per year in mild AD was used [23], and the predicted ADAS-cog decline was calculated based on two previously reported baseline-dependent empirical models of untreated AD [24,25].

## Discussion

In this three-year study of ChEI treatment in mild AD in a routine clinical setting, we found that patients’ cognitive ability and global performance were more preserved than their functional capacity. Using mixed-effects models, we found that a higher mean dose of ChEI (regardless of drug agent) and a lower level of education were predictors of more preserved longitudinal cognitive and functional abilities. Older individuals or those with less impaired IADL at baseline exhibited a slower progression rate in cognition, while younger age, living with a family member and higher cognitive status at baseline were predictors of less functional deterioration during the study. Inconsistent results regarding cognitive outcome were found between genders and APOE genotype. Regression models of expected decline in mild AD are presented.

There has been increased interest in disease progression in mild AD after new therapies such as solanezumab and souvenaid have shown positive results in this group exclusively. In the present study of mild AD, which includes all three ChEI agents, the three-year decline in ADAS-cog was 6.12 points and MMSE 3.10 points, suggesting a similar rate of change as in our previously described mild-to-moderate SATS group [10,26]. The US Food and Drug Administration has defined an improvement of at least four points in the ADAS-cog score as clinically significant (reported in [27,28]), and a MMSE change of three points has been suggested to indicate a clinically significant alteration in cognitive ability [29,30]. From the Alzheimer’s Disease Neuroimaging Initiative, Schneider *et al.*[31] reported that the ChEI-treated participants with mild AD deteriorated, on average, 9.25 points in ADAS-cog and 4.19 points in MMSE scores after two years in the study. The corresponding two-year mean decline in our cohort was considerably less, 3.81 and 2.20 points, respectively. An 18-month randomized controlled trial of tarenflurbil in mild AD showed 6.44 points ADAS-cog and 3.27 points MMSE mean deterioration in the placebo group (most patients were treated with ChEIs and/or memantine) [32]. As a comparison, our study showed, on average, lower deterioration after 18 months, 2.58 and 1.31 points, respectively. However, participants were included in these studies 1 year (median) and 20.5 months (mean) after AD diagnosis. Thus, these patients had been treated with ChEIs and/or memantine before the baseline, whereas the baseline in the SATS was the start of ChEI therapy. The slower disease progression observed in the SATS cohort might reflect the benefits of continuous ChEI therapy started almost immediately after AD diagnosis and the positive treatment response in the first months after initiation (baseline).

The current study of mild AD highlights the importance of not only assessing cognition at follow-ups, but also IADL capacity. A moderate linear relationship (r = 0.4 to 0.7) between cognitive and functional abilities has been observed in both untreated [33] and ChEI-treated [34] AD patients. Our study demonstrated that individuals with higher cognitive status at the start of ChEI therapy showed greater preserved IADL capacity over time and that those with a better IADL score at baseline exhibited a slower rate of cognitive decline. Patients showing a rapid functional decline have previously been shown to exhibit significantly greater impairment of cognitive ability at the time of AD diagnosis [11,35,36]. This relationship indicates that various aspects of the patients’ capabilities (cognitive, global, IADL) should be analyzed using multivariate methods, and these aspects should be interpreted simultaneously because a change in one ability might affect a change in another.

In the present ChEI-treated cohort with MMSE score 20 to 26, the rate of deterioration in IADL was faster than in cognitive ability and global performance. A marked decline in IADL capacity was also reported in the above-mentioned studies of mild AD [31,32]. Functional deficits have been observed as early as in amnestic mild cognitive impairment [37]. Three years after AD diagnosis, 70% to 85% of the remaining individuals in our mild SATS cohort could not carry out IADL tasks independently. The number of concomitant medications and usage of specific medications at baseline were not significant predictors of progression in IADL in the multivariate mixed-effects models in the current study, suggesting that comorbidity did not influence the functional outcome. These findings emphasize the importance of functional evaluations even for patients in the mild stage of AD in clinical routine, and raise the question of whether IADL is a better and more sensitive outcome measure than cognition in studies of potentially ‘disease-modifying’ therapies.

The rate of disease progression varies among AD patients. In particular, knowledge of prognostic factors in mild AD exclusively is strongly limited. This study shows that male gender, older age, a lower level of education, absence of the APOE ϵ4 allele, more preserved IADL ability and a higher mean dose of ChEI were significant predictors of slower cognitive decline. Younger age, lower educational level, living with a family member, better cognitive status and a higher ChEI dose were predictors of less functional deterioration. These findings are consistent with previous papers from the SATS including the entire mild-to-moderate cohort [11,38]. Using a mixed-models approach, Wilkosz *et al.*[39] also observed that male sex and older age were predictors of a slower longitudinal cognitive decline in AD. Several other studies also reported a more rapid rate of cognitive deterioration in younger individuals [40,41], but not all found that age was a significant predictor of decline [42]. It is reasonable to predict that when AD is manifest at younger ages, more hereditary early-onset and fast progression subtypes of the disease affect the outcome and could lead to a worse prognosis in mild AD as well. Our finding that a lower education level was related to a slower rate of cognitive deterioration was in agreement with a community-based study of mild dementia [43]. Lower education has also been reported to be associated with slower progression in mild-to-moderate AD [41,44]. Patients with low education level were assumed to have a reduced cognitive reserve and, therefore, would be more vulnerable to the effects of neurodegeneration. They would also be expected to perform worse on standardized cognitive tests that use a single threshold to identify dementia, such as the MMSE and ADAS-cog. These consequences of a lower education level might lead to an earlier manifestation of the typical symptoms of AD and detection of the disease. Furthermore, diagnosis and treatment might occur in an earlier stage, which may improve the results of AD therapy. The participants’ high level of education in the studies of mild AD from the United States [31,32] might be one reason for their faster cognitive decline compared with the SATS cohort. Impairment in several cognitive domains and its relation to level of education can already be observed several years before the clinical diagnosis of AD [45].

In the current study, the presence of at least one APOE ϵ4 allele was associated with a faster rate of cognitive decline when using the ADAS-cog scale but not the MMSE. Reports of the association of the ϵ4 allele with cognitive deterioration in the literature vary. Some studies report a positive relationship of APOE ϵ4 with more rapid cognitive decline in mild [46] and mild-to-moderate AD [47], while others report no significant influence on the rate of cognitive and functional progression [48,49], or that individuals with two ϵ4 alleles have a slower clinical course of AD [50]. Explorative analyses based on phase 2 data from passive immunization with the anti-Aβ antibody bapineuzumab found that non-carriers of the APOE ϵ4 allele had a better cognitive response compared with ϵ4-carriers after 18 months [12]; however, the phase 3 trials failed to replicate these results [51]. Yet, the treatment groups have been stratified into presence/absence of the APOE ϵ4 allele in some immunotherapy studies [52]. In our naturalistic SATS study including ChEI-treated patients with mild-to-moderate AD, APOE ϵ4-carriers exhibited both a lower cognitive response after six months of treatment and poorer long-term outcome after three years. On average, these individuals were younger at the start of ChEI treatment and had a higher level of education than the non-ϵ4 carriers [38]. A recent study of functional ability in amnestic mild cognitive impairment showed that patients carrying the APOE ϵ4 allele had more IADL deficits compared with non-carriers [37]. These findings highlight the importance of using an advanced multivariate statistical method, such as a mixed-effects model, to be able to take the potential predictive characteristics into account when analyzing disease progression [53]. The better response to drug agents observed in patients with no APOE ϵ4 alleles might not be a therapeutic effect, but possibly a slower natural decline.

Higher doses of ChEIs were related to a more favorable long-term cognitive and functional outcome in this study of mild AD. These results were also observed in the entire mild-to-moderate SATS cohort [11,38] as well as in a meta-analysis of randomized trials [54]. Moreover, a higher ChEI dose was associated with a delay in the need for nursing home placement [21,55]. A recent study from our group reported for the first time a significant relationship between a higher dose of ChEI and less need for home help services [56]. These effects and consequences show the importance of optimizing the ChEI dose for the individual patient with AD.

The advantages of the present three-year treatment study are the prospective, well-structured, regular six-month evaluations of a large cohort of continuously ChEI-treated mild AD patients from Swedish memory clinics. We enrolled more ordinary patients than the specially selected individuals usually included in clinical trials by using wide inclusion criteria, and allowing concomitant illnesses and medications. Moreover, the compliance to ChEI treatment in the SATS cohort was high [57]. The completion rate of 42% obtained for these participants from clinical practice was high compared with other AD extension or naturalistic studies [26]. The large attrition rate in all long-term AD studies may contribute to a better outcome for the patients remaining in the study, assuming that they benefit more from ChEI therapy. The SATS is an observational, non-randomized study, and has the limitations of an open-label design, and no untreated placebo group because of ethical concerns, similar to other AD therapy studies longer than six months. The choice of the ChEI agent and dose was left entirely to the individual physician’s discretion and professional judgment.

In the future, research efforts will focus on the development of AD starting in the early and pre-symptomatic stages. The timing of the start of therapy during the course of AD appears to be an essential factor that influences disease progression and the outcome of clinical trials. Currently, new pharmacological agents, such as immunotherapies, are being evaluated in randomized trials in addition to standard therapy (ChEI and/or memantine). Long-term observational studies of ChEI-treated AD patients exclusively in the milder stages are warranted to demonstrate realistic treatment expectations. The baseline-dependent prediction models of mild AD presented in the current study might be a useful tool to estimate the cognitive and functional outcomes that may be expected using ChEI monotherapy over time.

## Conclusions

In conclusion, an investigation of disease progression in different domains was described in this mild AD study. Our naturalistic SATS cohort exhibited a slower cognitive decline in comparison with those previously reported in the literature; however, the patients in those studies had been treated with ChEIs and/or memantine prior to baseline and had different socio-demographic characteristics. When comparing changes in scores between studies, it may be important to consider the point of time when ChEI therapy was initiated because the first months of positive response after initiation could imply a more favorable mean outcome over time. This study shows that socio-demographic and clinical factors such as age, years of education and the dose of ChEI, previously shown to affect the trajectories in mild-to-moderate patients, might alter the outcomes of various mild AD studies as well. The surprisingly fast deterioration in IADL compared with cognitive and global performance stresses the clinical importance of functional evaluations during the early stages of dementia. APOE genotype exhibited inconsistent results, which is noteworthy since this variable is used to select patients in AD trials. Information related to expected progression in various domains of the disease, and the influence of potential predictors, is necessary for evaluation of future treatments in mild AD. We present mathematical prediction models that could be used to assess the long-term outcomes of new therapies added to ChEI treatment.

## Abbreviations

AD: Alzheimer’s disease; ADAS-cog: Alzheimer’s Disease Assessment Scale-cognitive subscale; APOE: Apolipoprotein E; ChEI: Cholinesterase inhibitors; CI: Confidence interval; CIBIC: Clinician Interview-Based Impression of Change; IADL: Instrumental Activities of Daily Living Scale; MMSE: Mini-Mental State Examination; NINCDS-ADRDA: National Institute of Neurological and Communicative Disorders and Stroke and the Alzheimer’s Disease and Related Disorders Association; NSAIDs: Non-steroidal anti-inflammatory drugs; SATS: Swedish Alzheimer Treatment Study; SD: Standard deviation; SPSS: Statistical Package for the Social Sciences.

## Competing interests

The authors declare that they have no competing interests.

## Authors’ contributions

CW participated in the study, supervised the data collection, was responsible for the statistical design and for carrying out the statistical analyses, interpreted the results and drafted the paper. ÅKW participated in the study, assisted in analyzing and interpreting the data, and critically revised the manuscript. LM designed the study and critically revised the manuscript. All authors read and approved the final manuscript.
